# *Ascophyllum nodosum*-Based Biostimulants: Sustainable Applications in Agriculture for the Stimulation of Plant Growth, Stress Tolerance, and Disease Management

**DOI:** 10.3389/fpls.2019.00655

**Published:** 2019-05-29

**Authors:** Pushp Sheel Shukla, Emily Grace Mantin, Mohd Adil, Sruti Bajpai, Alan T. Critchley, Balakrishnan Prithiviraj

**Affiliations:** ^1^Marine Bio-products Research Laboratory, Department of Plant, Food and Environmental Sciences, Dalhousie University, Truro, NS, Canada; ^2^Research & Development, Acadian Seaplants Limited, Dartmouth, NS, Canada

**Keywords:** *Ascophyllum nodosum*, biostimulants, plant growth, stress tolerance, disease management

## Abstract

Abiotic and biotic stresses limit the growth and productivity of plants. In the current global scenario, in order to meet the requirements of the ever-increasing world population, chemical pesticides and synthetic fertilizers are used to boost agricultural production. These harmful chemicals pose a serious threat to the health of humans, animals, plants, and the entire biosphere. To minimize the agricultural chemical footprint, extracts of *Ascophyllum nodosum* (ANE) have been explored for their ability to improve plant growth and agricultural productivity. The scientific literature reviewed in this article attempts to explain how certain bioactive compounds present in extracts aid to improve plant tolerances to abiotic and/or biotic stresses, plant growth promotion, and their effects on root/microbe interactions. These reports have highlighted the use of various seaweed extracts in improving nutrient use efficiency in treated plants. These studies include investigations of physiological, biochemical, and molecular mechanisms as evidenced using model plants. However, the various modes of action of *A. nodosum* extracts have not been previously reviewed. The information presented in this review depicts the multiple, beneficial effects of *A. nodosum*-based biostimulant extracts on plant growth and their defense responses and suggests new opportunities for further applications for marked benefits in production and quality in the agriculture and horticultural sectors.

## Introduction

The global effects of negative climatic changes have manifested as desertification, increased atmospheric CO_2_ and temperature, soil salinization, and nutrient imbalances (e.g., mineral toxicity and deficiency) and have caused dramatic effects on agricultural production and the quality of crops (dos Reis et al., 2012). Such abiotic stresses have reduced the growth, development, productivity, and quality of plants and, in extreme conditions, resulted in death and local extinction of species (Matesanz et al., 2010; Anderson et al., 2011). Abiotic stresses are reported to have led to an average yield loss greater than 50% in most crops (Boyer, 1982; Vinocur and Altman, 2005). Rice yields declined 15% per 1°C rise in mean growing season temperature, measured from 1979 to 2003 (Peng et al., 2004). Additionally, changing climatic conditions can increase plant susceptibility to pathogens (West et al., 2012; Elad and Pertot, 2014), further increasing adverse growing conditions for plants.

The global amount of cultivable land available for agriculture is continuously shrinking due to urbanization and the adverse effects of climate change. In order to meet the ever-increasing demands of the growing human population, world food production must double by the year 2050 (Qin et al., 2011; Voss-Fels and Snowdon, 2016). To address the pressures associated with increasing agricultural productivity to subsequently meet the rising demands for food, producers have turned to excessive applications of synthetic (chemical) fertilizers and pesticides. These harmful chemicals pose both short- and long-term threats to the health of the entire biosphere (Damalas and Koutroubas, 2016). Therefore, an effective, biological-based alternative is required in order to reduce dependency on synthetic fertilizers and pesticides. Plant biostimulants are a new class of crop input, offering a potential alternative to traditional, agro-chemical inputs, and, in most cases, can reduce the application rates of synthetic fertilizers and pesticides by enhancing their efficacy (Calvo et al., 2014; Van Oosten et al., 2017; Yakhin et al., 2017).

According to the European Biostimulants Industry Council (EBIC), “plant biostimulants contain substance(s) and/or micro-organisms whose function when applied to plants or the rhizosphere is to stimulate natural processes to enhance/benefit nutrient uptake, nutrient efficiency, tolerance to abiotic stresses, and crop quality”^1^. The concept of biostimulants has been researched since 1933 (Yakhin et al., 2017) but has gained attention in more recent years as a potential solution to mitigate the negative impacts of a changing climate on agriculture. It should be noted that seaweed extracts are but one of the inputs that are classed as biostimulants.

**FIGURE 1:**
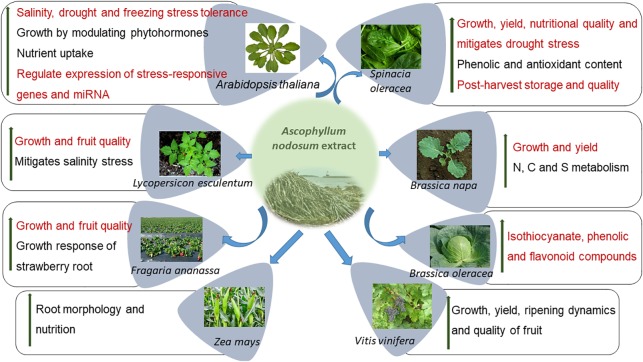
*Ascophyllum nodosum* extract (ANE) improves the growth of several crops by different modes of action.

Seaweeds are multi-cellular, macroscopic organisms found in coastal, marine ecosystems and are a rich source of polysaccharides, polyunsaturated fatty acids (PUFAs), enzymes, and bioactive peptides among others (Courtois, 2009; De Jesus Raposo et al., 2013; Ahmadi et al., 2015; Shukla et al., 2016; Okolie et al., 2018). In particular, inter-tidal seaweeds may be exposed to unfavorable conditions including extreme variations in temperature, salinity, and light. Seaweeds, as compared to terrestrial organisms, produce different stress-related compounds that are essential for their survival in these environments (Shukla et al., 2016). As such, selected seaweed resources are important sources of plant biostimulants and are widely used to promote agricultural productivity (Khan et al., 2009; Sharma et al., 2014; du Jardin, 2015; Van Oosten et al., 2017). The most widely researched seaweed, used as a source for industrial and commercial plant biostimulants, is the brown, inter-tidal seaweed *Ascophyllum nodosum*. Various commercial extracts from *A. nodosum* have been demonstrated to improve plant growth, mitigate some abiotic and biotic stresses while also improving plant defenses by the regulation of molecular, physiological, and biochemical processes. Of all sources of seaweed-based biostimulants, those manufactured from *A. nodosum* are perhaps the best studied with various modes of action being proposed (Figure 1). This review focuses on accumulating current knowledge of the bioactive compounds presents in *A. nodosum* extracts and their modes of action in promoting plant growth in the presence of abiotic and biotic stresses.

## Modes of Extraction

Various commercial entities utilize different, proprietary extraction (hydrolysis) procedures for the production of seaweed-based biostimulants in either liquid or soluble powder form (Kadam et al., 2013; Michalak and Chojnacka, 2015). Different extraction methods have been cited in the literature using both dry and wet biomass (Chojnacka et al., 2015; Michalak and Chojnacka, 2015; Bleakley and Hayes, 2017). The bioactivity and composition of *A. nodosum* biostimulants are not all identical and are indeed dependent on the extraction methods employed (Goñi et al., 2016).

### Water-Based Extractions

The name of this extraction method is indicative of the process: biostimulatory compounds are harvested by blending and hydrating dried seaweed meal in the presence of water (Sharma et al., 2014). The solid residues are separated using different filtration methods based on the end use of the biostimulant. Biostimulants prepared using this method are reportedly rich in phytohormone-like activity (Blunden and Wildgoose, 1977; Crouch and van Staden, 1993).

### Acid Hydrolysis

In this method, freshly chopped *Ascophyllum* biomass was treated with sulfuric acid or hydrochloric acid at 40–50°C for 30 min (Sharma et al., 2014). It was reported that acid hydrolysis removed complex phenolic compounds and increased de-polymerization of polysaccharides (Flórez-Fernández et al., 2018). This method is generally used for the extraction of fucose-containing sulfated polysaccharides (Ale et al., 2012; Flórez-Fernández et al., 2018). Sulfated algal polysaccharides are a class of bioactive compounds in algal extracts that promote plant growth (Fry et al., 1993; Paulert et al., 2009; Shukla et al., 2016). Marais and Joseleau (2001) purified fucoidans from *A. nodosum* by acid hydrolysis. AZAL5^®^ is a commercially available biostimulant manufactured from *A. nodosum*, which is extracted through acid hydrolysis (Jannin et al., 2013).

### Alkaline Hydrolysis

Alkaline hydrolysis is perhaps the most widely used industrial hydrolysis process for the production of an extract from *A. nodosum* (Craigie, 2011; Sharma et al., 2014; Flórez-Fernández et al., 2018). This method involves extracting *A. nodosum* biomass in NaOH or KOH solutions, at “relatively low” temperatures, between 70 and 100°C. This process breaks down complex polysaccharides into smaller, lower-molecular-weight oligomers. The alkali treatment of *Ascophyllum* biomass produces novel compounds that are not initially present within the seaweed biomass. These compounds are a result of the interaction between the hydrolysis chemicals (KOH) and constituents of the brown seaweed tissues—the result of degradation, rearrangement, condensation, and base-catalyzed synthetic reactions (Craigie, 2011). Alkali treatments of brown seaweed biomass also act on polyphenols in the tissue to produce a complex array of reaction products, which are dependent on the hydroxylation pattern of the original polyphenol (Craigie, 2011). Maxicrop^®^ (United States), Seasol^®^ (Australia), and Acadian^®^ (Canada) are major commercial biostimulants that are manufactured using an alkali extraction process of *Ascophyllum.*

### Microwave-Assisted Extraction

Microwave-assisted extraction (MAE) is suggested to be an eco-friendly extraction method for the manufacture of biostimulants from algal biomass, as compared to other solvent-based extraction procedures (Michalak et al., 2015b). In this method, slurry prepared from dried algal biomass, in either water or microwave-supported solvent, is heated by microwave energy to extract bioactive compounds (Magnusson et al., 2017; Flórez-Fernández et al., 2018). Microwave heating is based on dipole polarization and the ionic conduction of the seaweed-derived bioactive compounds into the solvent (Lucchesi et al., 2004; Flórez et al., 2015). This extraction method is favored for its efficient use of time and materials given the resultant selective extraction of carbohydrates, proteins, and other fractions (Eskilsson and Björklund, 2000; Routray and Orsat, 2012). Additionally, this extraction method was found to improve the efficiency of extraction by controlling sub-critical properties of the solvent (Routray and Orsat, 2012; Magnusson et al., 2017). MAE has been used to extract fucoidan, sodium alginate, sugars, and phenolic compounds from *A. nodosum* (Yuan and Macquarrie, 2015a,b; Yuan et al., 2018).

### Ultrasound-Assisted Extraction

Ultrasound-assisted extraction (UAE) is reported as another eco-friendly method for obtaining bioactive compounds from algal biomass. Ultrasound waves are high frequency (greater than 20 kHz), which transmit through solid, liquid, and gas media by rarefactions (largest distance between wave particles) and compression (smallest distance between wave particles) (Kadam et al., 2013). Ultrasound waves were reported to facilitate the release of bioactive compounds from a variety of seaweed biomass by cavitation within the extraction solvent (Kadam et al., 2013, 2015a). When cavitation (i.e., the formation and eventual implosion of empty spaces or bubbles) occurs near seaweed cell walls, the transfer of compounds from the cell to the solvent is facilitated following cellular breakdown (Kadam et al., 2013, 2015a). UAE is a cost-effective and efficient method of extraction when compared to other extraction protocols based on the limited equipment needed and the vast array of solvents that can be used (Kadam et al., 2013). Kadam et al. (2015b, c) optimized the extraction procedure for the isolation of numerous bioactive compounds, including laminarin from both *A. nodosum* and *Laminaria hyperborea*.

### Enzyme-Assisted Extraction

Enzyme-assisted extraction (E-AE) is an eco-friendly and efficient extraction method as there are no solvents required by the process (Kadam et al., 2013). The efficiency of the extraction lies in the enzymatic degradation of the complex molecules present in the seaweed cell walls (Wijesinghe and Jeon, 2012; Kadam et al., 2013). Enzymes are chosen strategically based on specific molecules digested from seaweed biomass in order to release the bioactive compounds (Wijesinghe and Jeon, 2012). Various carbohydrate-degrading enzymes and proteases such as Viscozyme, Cellucast, Termamyl, Ultraflo, carrageenanase, agarase, xylanase, Kojizyme, Neutrase, Alcalase, and Umamizyme are commonly used for the extraction of bioactive compounds from seaweeds (Ahn et al., 2004; Heo et al., 2005; Wijesinghe and Jeon, 2012; Kadam et al., 2013). The application of hydrolytic enzymes converts the water-insoluble chemical components of selected seaweed biomass to water-soluble products, thus eliminating the problem of water solubility of bioactive compounds (Wijesinghe and Jeon, 2012; Kadam et al., 2013). To date, there are no publications regarding the extraction of bioactive compounds from *A. nodosum* using E-AE. It has been reported that E-AE extracts of other seaweeds (i.e., *Ecklonia cava*, *Ishige okamurae*, *Sargassum fulvellum*, *S. horneri*, *S. coreanum*, *S. thunbergii*, and *Scytosiphon lomentaria*) showed higher antioxidative activity, as compared to commercial antioxidants (Heo et al., 2005). In the future, this method might be applied for the extraction of bioactive compounds from *A. nodosum*.

### Super-Critical Fluid Extraction

The super-critical fluid extraction (SFE) method is yet another eco-friendly method of bioactive extraction from seaweeds, based on the lack of toxic solvents required for extraction (Herrero et al., 2010; Michalak et al., 2016b). This method protects the parent seaweed material against thermal or biochemical degradation of the bioactive compounds (Herrero et al., 2010; Michalak et al., 2015a; da Silva et al., 2016). Bioactive compounds are extracted in the presence of super-critical organic solvents (often CO_2_, based on its critical conditions, availability, and high diffusivity when mixed with ethanol; Herrero et al., 2010; Kadam et al., 2013). The higher penetration of the solvent into the seaweed material during SFE results in better mass transfer between solvent phases (Michalak et al., 2015a; Messyasz et al., 2018). Michalak et al. (2016a) showed that super-critical extracts of *A. nodosum* enhanced the growth and development of winter wheat.

### Pressurized Liquid Extraction

Pressurized liquid extraction (PLE) was first reported by Richter et al. (1996). In this method, extraction was carried out under high pressure (3.5–20 MPa) and temperature (50–200°C) (Kadam et al., 2013). The high pressure elevated the temperature of solvents above their boiling point, facilitating bioactive compound extraction by increasing the solubility of complex algal molecules and increasing mass transfer rate (Kadam et al., 2013; Michalak and Chojnacka, 2015). PLE is a faster extraction method compared to other methods; however, Tierney et al. (2013) showed that the application of high temperatures (50–200°C) and pressures (500–3,000 psi) during extraction did not enhance the antioxidant activities of extracts from *A. nodosum*, *Pelvetia canaliculata*, *Fucus spiralis*, and *Ulva intestinalis*, as compared to extracts from the traditional solid liquid extraction method.

In addition to the aforementioned techniques, different extraction methods were also used in combination for extracting protein from *A. nodosum* extract. Kadam et al. (2017) combined ultrasound pretreatment with acid and alkali hydrolysis and, more simply, combined acid and alkali hydrolysis to extract protein from *A. nodosum*. The initial treatment of the *A. nodosum* with acid followed by a treatment with alkali was found to be the most efficient method among all methods investigated (Kadam et al., 2017). Similarly, combining enzymatic hydrolysis with alkaline extraction also increases the efficiency of protein extraction in *Palmaria palmata* (Maehre et al., 2016). The combination of extraction methods has not yet been employed for the extraction of biostimulants from *A. nodosum* for plant growth, creating opportunities for the future.

## Ascophyllum nodosum

*Ascophyllum nodosum* is commonly known as rockweed, and is abundantly distributed throughout the northwest coast of Europe and the northeastern coast of North America (Moreira et al., 2017). Craigie (2011) reviewed the unique characteristics of *A. nodosum* as a prominent source for the production and synthesis of biostimulants. One unique feature of *A. nodosum* is its mutualistic association with the fungal endophyte *Mycosphaerella ascophylli* (Fries and Thorén-Tolling, 1978; Fries, 1979; Garbary and Gautam, 1989; Craigie, 2011). *M. ascophylli* protects *A. nodosum* from desiccation (Garbary and London, 1995). Further, results published by Prithiviraj et al. (2011) showed that *M. ascophylli*-derived fungal sterols present in the ethyl acetate extract of *A. nodosum* mitigated salinity stress in plants.

Based on the review published by Van Oosten et al. (2017), nearly 47 companies worldwide are currently involved in manufacturing extracts from *A. nodosum* for agricultural and horticultural applications. *A. nodosum* is a rich source of various bioactive phenolic compounds such as phlorotannins and unique polysaccharides, i.e., alginic acid (28%), fucoidans (11.6%), mannitol (7.5%), and laminarin (4.5%) (Ragan and Jensen, 1977; Holdt and Kraan, 2011; Yuan and Macquarrie, 2015a; Moreira et al., 2017). Commercially dried and milled, *A. nodosum* meal is reported to contain carbohydrate (44.7 ± 2.1%), ash (18.6 ± 0.9%), protein (5.2 ± 0.2%), lipids (3.0 ± 0.1%), phenolics (1.4 ± 0.2%), and other compounds (13.6%) (Yuan and Macquarrie, 2015b; Moreira et al., 2017). Some of these compounds showed considerable seasonal variation (Parys et al., 2009; Craigie, 2011). The bioactive compounds present in *A. nodosum* were extracted and utilized to promote plant growth according to Van Oosten et al. (2017).

## ANE Improves Fruit Quality, Plant Growth, and Yield

Commercial, hydrolyzed extracts from *A. nodosum* (ANE) have been repeatedly demonstrated to exhibit growth-stimulating activities in treated plants, when applied repeatedly at very low doses, and are referred to as “biostimulants” (Sharma et al., 2014; Van Oosten et al., 2017). Table 1 lists publications on the growth-promoting activities of commercial extracts of *A. nodosum*. The applications of different extracts of *A. nodosum* are repeatedly demonstrated to improve the growth and productivity of crops through various modes of action (Figure 1).

**Table 1:** List of extracts manufactured from *A. nodosum* biomass that were reported to improve plant growth.

S. No.	Extract	Crop	Function	References
1	GA14^®^ (Goemar, France)	*Spinacia oleracea*	Foliar spray improved total fresh biomass	Cassan et al., 1992
2	Maxicrop^®^ Original	Tomato	Higher chlorophyll content in sprayed plants	Whapham et al., 1993
3	Maxicrop^®^	*Capsicum annuum*	Improved yield and quality	Eris et al., 1995
4	Goemar^®^	*Citrus unshiu*	Early maturation of fruit	Fornes et al., 1995
5	*A. nodosum* extract	Kiwi fruit	Improved fruit growth, weight, and maturation	Chouliaras et al., 1997
	*A. nodosum* extract	Tomato, dwarf French bean, wheat, barley, maize	Enhanced leaf chlorophyll level	
6	Acadian^®^ (Acadian Seaplants)	*Vitis vinifera*	Improved yield and fruit quality	Norrie et al., 2002
7	Acadian^®^ (Acadian Seaplants)	*Poa pratensis*	Improved shelf life and transplant rooting	Zhang et al., 2003
8	Maxicrop^®^, Proton^®^, Algipower^®^	*Vitis vinifera*	Improved copper uptake of grapevine	Turan and Köse, 2004
9	Goemar^®^	Clementine Mandarin and Navelina Orange	Increased productivity and yield	Fornes et al., 2002
10	*A. nodosum* extract	*Arabidopsis thaliana*	Improved plant growth by modulation of concentration and localization of auxin	Rayorath et al., 2008
11	*A. nodosum* extract	*Hordeum vulgare*	Induced gibberellic-acid-independent amylase activity in barley and promoted seed germination	Rayorath et al., 2008
12	Goëmar BM 86^®^	Apple	Improved the fruit quality of apple and have high nitrogen content	Basak, 2008
13	Acadian^®^ Marine Plant Extract Powder (AMPEP)	*Kappaphycus striatum*	AMPEP improves micro-propogation	Hurtado et al., 2009
14	*A. nodosum* extract	*Olea europaea*	Showed increased tree productivity and improved their nutrition status and oil quality parameters	Chouliaras et al., 2009
15	Alge^®^	*Citrullus lanatus*	Application of extract showed increased growth parameters and yield responses	Abdel-Mawgoud et al., 2010
16	Actiwave^®^	Strawberry	Increases fruit yield and quality and acts as iron chelator	Spinelli et al., 2010
17	Acadian^®^ (Acadian Seaplants)	*Spinacia oleracea*	Enhances phenolic antioxidant content of Spinach	Fan et al., 2011
18	AMPEP	*Ulva lactuca*	Reduces ionic liquid induced oxidative stress in *Ulva lactuca*	Kumar et al., 2013
19	*A. nodosum extract*	*Medicago sativa*	Improves root colonization of rhizobial symbionts	Khan et al., 2012
20	*A. nodosum* extract	Strawberry	Improved plant growth, fruit quality and microbial growth	Alam et al., 2013
21	Super Fifty^®^, Ecoelicitor^®^	Lettuce; Oilseed rape	Enhanced plant growth and tolerance to biotic and biotic stresses	Guinan et al., 2012
22	Acadian^®^ (Acadian Seaplants)	*Spinacia oleracea*	Improved yield and nutritional quality	Fan et al., 2013
23	Acadian^®^ (Acadian Seaplants)	*Spinacia oleracea*	Improves phenolics and antioxidant content of spinach	Fan et al., 2013
24	Alga Special (AS)	*Vitis vinifera*	Improved vegetative growth	Popescu and Popescu, 2014
25	AZAL5	*Brassica napus*	Promotes plant growth and nutrient uptake	Jannin et al., 2013
26	AlgaeGreen^®^	*Brassica oleracea*	Enhanced biosynthesis of secondary metabolites	Lola-Luz et al., 2013
27	Acadian^®^ (Acadian Seaplants)	*Spinacia oleracea*	Preharvest ANE application enhanced post-harvest storage quality of spinach	Fan et al., 2014
28	Acadian^®^ (Acadian Seaplants)	Carrot	Promote plant growth and root yield in carrot associated with increased root-zone soil microbial activity	Alam et al., 2014
29	Stella Maris^TM^	*Calibrachoa hybrida*	Increased biosynthesis of secondary metabolites and enhanced antibacterial and antifungal properties of *C. hybrida* extract	Elansary et al., 2016a
	*A. nodosum* extract	*Vitis vinifera*	Improved growth, yield, berry quality attributes, and leaf nutrient content of grapevines	Sabir et al., 2014
30	Premium liquid seaweed	*Allium cepa*	Improved vegetative growth and yield of onion	Hidangmayum and Sharma, 2017
31	Seaweed extract	*Zea mays*	Promotes root morphology and plant nutrition	Ertani et al., 2018
32	Acadian^®^ (Acadian Seaplants)	*Vitis vinifera*	Foliar spray has a positive effect on ripening dynamics and fruit quality	Frioni et al., 2018
33	Rygex^®^, Super fifty^®^	*Solanum lycopersicum*	Increased plant growth and fruit quality and mitigates salinity stress in tomato plants	Di Stasio et al., 2018
34	Seaweed extract	*Spinacia oleracea*	Improved growth, quality, and nutritional value of spinach grown under drought conditions	Xu and Leskovar, 2015
35	Seasol^®^	*Fragaria ananassa*	Increased growth response of strawberry root	Mattner et al., 2018

### Fruit Quality

A foliar spray of *A. nodosum* improved fruit quality of watermelons, apples, olives, and grapes (Basak, 2008; Chouliaras et al., 2009; Abdel-Mawgoud et al., 2010; Frioni et al., 2018). Foliar application of ANE also increased the ripening rate of grapes (Norrie et al., 2002; Sabir et al., 2014; Frioni et al., 2018) and increased oil content and consistency of fruit maturation in olive (Chouliaras et al., 2009).

### Nutrient Acquisition, Accumulation, and Biosynthesis

*Ascophyllum nodosum* was reported to improve both the growth and productivity of agricultural crops by increasing nutrient availability and uptake (Crouch and van Staden, 1993; Khan et al., 2009; Craigie, 2011; Sharma et al., 2014; Van Oosten et al., 2017). Several publications indicated that a foliar application of ANE to the leaves of *Vitis vinifera*, after full bloom, increased the nutrient content of grapevines, specifically the accumulation of anthocyanins and phenolics (Norrie et al., 2002; Sabir et al., 2014; Frioni et al., 2018). Two commercial extracts of *A. nodosum*, Rygex^®^ and Super Fifty^®^, enhanced the macronutrient (N, P, K, Ca, S) and micronutrient (Mg, Zn, Mn, Fe) contents of tomato fruits (Di Stasio et al., 2018). Similarly, olive plants (*Olea europaea*) treated with ANE showed a higher uptake of K, Fe, and Cu (Chouliaras et al., 2009). When applied at a rate of 0.1% (v/v), AZAL5^®^, a commercial seaweed extract, improved root and shoot growth of rapeseed (*Brassica napus*) by stimulating nitrogen and sulfate accumulation (Jannin et al., 2013). Microarray analysis revealed that *B. napus* plants treated with AZAL5^®^ showed differential regulation of 724 and 298 genes in shoots and roots, respectively, after 3 days of treatment, while 612 and 439 genes were differentially regulated in the shoots and roots, respectively, after 30 days of treatment (Jannin et al., 2013). Treatment with AZAL5^®^ increased the nitrate uptake of *B. napus* by inducing the expression of *BnNRT1.1* and *BnNRT1.2* genes, known to be involved in nitrate assimilation and amino acid metabolism. Similarly, plants treated with AZAL5^®^ showed higher sulfate accumulation by the induction of *BnSultr1.1* and *BnSultr1.2* genes (Jannin et al., 2013). Commercial extracts Maxicrop^®^, Proton^®^, and Algipower^®^ were also reported to improve the nutrient uptake of grapevines (*V. vinifera*) (Turan and Köse, 2004).

*Ascophyllum nodosum* extracts enhanced the growth of leafy vegetables such as spinach (*Spinacia oleracea*) and lettuce (*Lactuca sativa*) (Cassan et al., 1992; Moller and Smith, 1998; Fan et al., 2013; Chrysargyris et al., 2018). A root-drench application of ANE induced the expression of glutamine synthetase in spinach (Fan et al., 2013), which is responsible for the conversion of inorganic ammonium to organic glutamine, and also plays an important role in nitrogen metabolism and assimilation (Oliveira et al., 2002). In addition to this, root application of ANE induced the expression of nitrate reductase, an important enzyme involved in nitrogen assimilation, which catalyzes the conversion of nitrate to nitrite (Fan et al., 2013). Taken together, these results suggest that ANE plays an important role in plant growth by enhancing nutrient uptake through the regulation of genes involved in nutritional acquisition. Pre-harvest treatment with 1.0 g/L of ANE through a root-drench improved the quality and nutrient content of spinach during post-harvest storage (Fan et al., 2014). The foliar application of 1% Biopost AG200^®^ liquid seaweed extract (Cofuna, France) biweekly for 5 weeks enhanced the relative growth and quality (post-harvest) of lettuce grown under K-deficient conditions by increasing antioxidant activity (Chrysargyris et al., 2018).

Pre-harvest root treatment by ANE (Acadian^®^) was reported to have a profound effect in reducing post-harvest losses by reducing lipid peroxidation (Fan et al., 2014). The results presented by Fan et al. (2011) reported that an application of ANE increased antioxidants and stimulated phenolic compound biosynthesis in spinach. Furthermore, the ANE-induced biosynthesis of phenolic antioxidants in spinach, when provided as a feed intake, protected *Caenorhabditis elegans* against oxidative and thermal stress (Fan et al., 2011). Similarly, the provision of Tasco^®^-Forage, a feed supplement (air dried and milled *A. nodosum*), improved non-enzymatic antioxidant compounds such as α-tocopherol, ascorbic acid, and β-carotene in turf and forage grasses (Allen et al., 2001). Tasco^®^-Forage also induced the activity of the enzymes superoxide dismutase (SOD), glutathione reductase, and ascorbate peroxidase (APX) in forage grasses (Allen et al., 2001). AlgaeGreen^®^, a commercial *A. nodosum* extract, increased the yield and secondary metabolite content of cabbage (*Brassica oleraceae*) (Lola-Luz et al., 2013). Treatment with ANE significantly enhanced vegetative growth as well as the biosynthesis of bioactive molecules such as phenolics and flavonoids of *Calibrachoa hybrid*, a medicinal plant (Elansary et al., 2016a). ANE-induced biosynthesis of secondary metabolites further enhanced the antifungal and antibacterial activity of the extract of *Calibrachoa* (Elansary et al., 2016a).

A root application of 1.0 g/L ANE was reported to induce the accumulation of transcripts of betaine aldehyde dehydrogenase (*BADH*) and choline mono-oxygenase (*CMO*) in spinach grown *in vitro* (Fan et al., 2013). These enzymes are known to catalyze a two-step pathway involved in the biosynthesis of glycine betaine in plants. Glycine betaine, an amphoteric quaternary ammonium compound, is an efficient, compatible solute that protects plants against environmental stresses (Sakamoto and Murata, 2002). The *A. nodosum* extract used was shown to contain quaternary ammonium compounds such as glycine betaine, δ-aminovaleric acid betaine, γ-aminobutyric acid betaine, and laminine (Blunden et al., 1985, 1996; Whapham et al., 1993; MacKinnon et al., 2010).

Hurtado and Critchley (2018) reviewed the biostimulant effect of *Ascophyllum* (Acadian^®^) Marine Plant Extract Powder (AMPEP) in increasing the cultivation and micro-propagation of the commercially important seaweed, *Kappaphycus alvarezii*. The application of AMPEP, a product derived from *A. nodosum*, improved the biomass cultivation of *K. alvarezii* (Marroig et al., 2016). The results of Tibubos et al. (2017) showed that AMPEP induced the direct formation of axes in new plantlets of *K. alvarezii.* These results provide clear evidence that the *Ascophyllum*-derived extract can potentiate growth of commercially important seaweed crops.

## ANE Improves Plant Growth by Regulating Phytohormone Biosynthesis in Plants

Phytohormones are low-molecular-weight compounds produced in very small quantities that regulate several physiological and developmental processes in plants (Wally et al., 2013; Wani et al., 2016). The most common phytohormones include auxins (IAA), cytokinins (CK), abscisic acid (ABA), gibberellic acid (GA), ethylene, jasmonic acid (JA), and salicylic acid (SA) (Wani et al., 2016). One reported growth-promoting effect of ANE was ascribed to the presence of a variety of “phytohormone-like substances” (Stirk and Van Staden, 1997; Khan et al., 2009; Craigie, 2011; Sharma et al., 2014; Battacharyya et al., 2015).

There is a wide variation in auxin content in *A. nodosum* extracts reported in the literature. *A. nodosum* was reported to have a high concentration of indole acetic acid (IAA), approximately 50 mg/g of dry extract (Kingman and Moore, 1982; Khan et al., 2009), whereas Maxicrop^®^, a different commercial product also prepared from *A. nodosum*, contained 6.63 mg of IAA per gram of dried powder (Sanderson et al., 1987). By using ultra-performance liquid chromatography–electrospray tandem mass spectrometry, Wally et al. (2013) confirmed the presence of 25–35 ng of IAA per dry gram of the extract they tested. This variation in auxin content is likely to be a function of the method of extraction and processing, as well as the geographical location of the raw material harvested including any possible seasonal variation (Stirk and Van Staden, 1996; Wally et al., 2013).

SAURs (small auxin-up RNAs) are a group of small auxin-induced RNAs that reportedly play an important role in cellular, physiological, and developmental processes (Ren and Gray, 2015). The expression of *SAUR33*, *SAUR59*, and *SAUR71* were up-regulated by the foliar application (0.2%) of commercially available neutral and alkaline extracts from *A. nodosum*, while *SAUR1* and *SAUR50* were down-regulated by both extracts (Goñi et al., 2016). Buggeln and Craigie (1971) reported biologically active homologs of auxin-like compounds in alkaline hydrolysates of *A. nodosum*. Bioactive compounds present in a methanolic fraction of this commercial ANE elicited plant growth by enhancing root tip growth and showed higher GUS expression in the *DR5*: GUS transgenic line of *Arabidopsis thaliana* (Rayorath et al., 2008). These findings strongly suggested that the organic fraction of ANE regulated auxin activity in ANE-treated plants through the regulation of auxin-responsive promoter elements (*AuxRE*) (Rayorath et al., 2008).

Cytokinins are derivatives of adenines that possess either an isoprenoid or aromatic side chain at the N6 position (Frébort et al., 2011). Previously published reports demonstrated that various cytokinins and “cytokinin-like compounds” were the most abundant plant growth regulators present in commercial extracts of *A. nodosum* (Stirk and Van Staden, 1997; Khan et al., 2011; Wally et al., 2013). Maxicrop^®^ was reported to contain a complex of cytokinins including zeatin, di-hydrozeatin, iso-petenyladenine, and iso-petenyladenosine (Sanderson et al., 1987). Stirk and Van Staden (1996) investigated the cytokinin activity of the commercial seaweed extract Seamac^®^ by evaluating its effect on soybean callus, where Seamac^®^ induced maximum soybean callus formation. Khan et al. (2011) and Wally et al. (2013) showed that a root application of an alkaline extract of *A. nodosum* resulted in the activation of the cytokinin-responsive promoter *ARR5*. The application of this commercial seaweed extract to *A. thaliana* showed a higher concentration of CK and ABA, coupled with a reduction in IAA levels. This observation helps to explain the varied mechanisms of actions behind higher vegetative plant growth and the reduction in the length of primary roots (Wally et al., 2013).

Wally et al. (2013) showed that the application of an ANE increased cytokinin concentrations in the tissues of *A. thaliana*, particularly *trans*-zeatin-type and *cis*-zeatin-type cytokinins. The first step of cytokinin biosynthesis involves the transfer of an isoprenoid molecule to adenine by isopentenyl transferases (IPTs). ANE applications induced the expression of *IPT3*, *IPT4*, and *IPT5* in *A. thaliana*, while the expressions of *IPT2* and *IPT9* were unchanged (Wally et al., 2013). In this study, ANE also regulated the transcript levels of cytosolic and mitochondrial *IPT*s (*IPT3*, *IPT4*, and *IPT5*) and induced the production of isopentenyl-type cytokinins *via* the mevalonate (MVA) pathway (Frébort et al., 2011; Wally et al., 2013). Similar to the expression pattern of *IPT3*, *IPT4*, and *IPT5*, the expression of CK hydroxylases (*CYP735A2*), which catalyze the biosynthesis of *trans*-zeatin, was higher in ANE-treated *A. thaliana* plants (Takei et al., 2004; Wally et al., 2013). Furthermore, the ANE treatment suppressed the expression of genes involved in cytokinin degradation (Wally et al., 2013). The accumulation of cytokinin oxidase 4 (*CKX4*), involved in cytokinin catabolism, was reduced in ANE-supplemented Arabidopsis plants. This suggests that ANE applications induced a higher metabolic production of cytokinins within treated plant tissues by differentially regulating cytokinin metabolism. High cytokinin content in plants was found to delay senescence (Gan and Amasino, 1995; Lim et al., 2003). Wally et al. (2013) showed that the ANE application retarded senescence in treated Arabidopsis by increasing the endogenous cytokinin content. This result was further supported by the strong inhibition of expression of Senescence Associate Gene 13 (*SAG13*) in plants treated with ANE (Wally et al., 2013).

The root application of ANE modulated the expression of genes involved in GA biosynthesis and thus resulted in a higher accumulation of *GA24* (Wally et al., 2013). Similarly, a foliar application of 0.2% ANE on Arabidopsis leaves also regulated the expression of the GA-responsive genes *GASA1* and *GASA4*, after 1 week of treatment (Goñi et al., 2016). This published evidence concluded that ANE treatment regulated endogenous phytohormone levels and possibly their ratios to one another within treated plant tissues by modulating the expression of genes involved in their biosynthesis. Subsequently, the modulation of gene expression improved plant growth and development.

## ANE Mitigates Abiotic Stresses in Plants

Plants, being sessile, are relentlessly challenged by a variety of environmental stresses that limit their growth and productivity (Agarwal et al., 2013; Shukla et al., 2016). Due to the complex metabolic pathways involved in stress tolerance, limited success has been achieved in generating stress-tolerant crops through genetic engineering (Agarwal et al., 2013; Mickelbart et al., 2015). Another sustainable approach to improve stress tolerance in plants is the use of extracts from *A. nodosum*. Table 2 summarizes studies published on the effect of ANE on plants under abiotic stress.

**Table 2:** List of the different extracts from *A. nodosum* conferring salinity stress tolerance in various crops.

S. No.	Extract	Crop	Function	References
1	Goemar	*Citrus unshiu*	Early maturation of fruit	Fornes et al., 1995
2	Acadian^®^	*Agrostis stolonifera*	Increased heat stress tolerance by seaweed-extract-based cytokinin	Zhang and Ervin, 2008
3	Acadian^®^	*A. thaliana*	Lipophilic component of *A. nodosum* extract enhanced freezing tolerance by protecting membrane integrity and modulates the expression of freezing stress responsive genes	Rayirath et al., 2009; Nair et al., 2012
4	Stimplex^®^	*Citrus sinensis*	Improves drought stress tolerance and maintains shoot growth under drought conditions	Spann and Little, 2011
5	Super Fifty, Ecoelicitor	Lettuce; oilseed rape	Enhanced plant growth and tolerance to biotic and abiotic stresses	Guinan et al., 2012
6	AMPEP	*Ulva lactuca*	Reduces ionic liquid induced oxidative stress in *Ulva lactuca*	Kumar et al., 2013
7	Stella Maris^TM^	*Calibrachoa hybrida*	Increased biosynthesis of secondary metabolites and enhanced antibacterial and antifungal properties of *C. hybrida* extract	Elansary et al., 2016a
8	Stimplex^®^	*Spiraea nipponica*, *Pittosporum eugenioides*	Improve drought tolerance by inducing phytochemical and antioxidant contents	Elansary et al., 2016b
9	Stella Maris^TM^	*Paspalum vaginatum*	Higher plant growth under prolonged irrigation and saline conditions by regulating osmotic adjustment and antioxidant defense system	Elansary et al., 2017
10	Algea^®^	*A. thaliana*	Acclimate plant to the drought stress by improving photosynthesis and water use efficiency and by regulating stress-responsive gene expression	Santaniello et al., 2017
11	*A. nodosum* extract	*Lycopersicon esculentum*	Enhanced tolerance to drought stress in tomato plants by modulating expression of dehydrins	Goñi et al., 2018
12	*A. nodosum* extract	*Phaseolus vulgaris*	Increased tolerance to the drought stress by affecting proline metabolism	Carvalho et al., 2018
13	Acadian^®^	*Glycine max*	Improve drought tolerance by modulating expression of stress-responsive gene	Shukla et al., 2018b
14	Seaweed extract	*Spinacia oleracea*	Improve growth, quality, and nutritional value of spinach grown under drought conditions	Xu and Leskovar, 2015

### ANE Improves Salinity Tolerance in Plants

Soil salinity is a global problem, affecting over 800 million hectares of land, resulting in massive impacts on agricultural productivity (Shrivastava and Kumar, 2015; Ferchichi et al., 2018). Mild salinity stress causes physiological drought in plants, impairing cell–water relations, inhibiting cell expansion, and, consequently, reducing growth rate (Hasegawa et al., 2000a). Long-term exposure to high salinity causes ionic stress by disturbing the homeostasis of intracellular ions, which results in membrane dysfunction and attenuation of metabolic activity and secondary effects, inhibiting growth, and inducing cell death (Hasegawa et al., 2000b; Yadav et al., 2012; Hasegawa, 2013; Shukla et al., 2011, 2015). Salinity induces both ionic and osmotic stresses, thus reducing plant growth and productivity (Agarwal et al., 2013). Plants have developed strategies to adapt to salinity stress at molecular, biochemical, and physiological levels (Agarwal et al., 2013; Hasegawa, 2013; Ferchichi et al., 2018).

Studies revealed that the application of various forms of ANE improved salinity stress tolerance in Arabidopsis, tomato (*Solanum lycopersicum*), passion fruit (*Passiflora edulis*), and avocado (*Persea americana*) (Jithesh et al., 2012, 2018; Bonomelli et al., 2018; Di Stasio et al., 2018; Jolinda et al., 2018; Shukla et al., 2018a). Rygex^®^ and Super Fifty^®^, both commercial extracts of *A. nodosum*, boosted the accumulation of minerals, antioxidants, and essential amino acids in tomato fruits grown under salinity stress (Di Stasio et al., 2018). Salinity stress reduced both the growth and yield of avocado by almost 50% (Alvarez-Acosta et al., 2018; Bonomelli et al., 2018). The application of *A. nodosum*-based extracts reportedly alleviated the effects of salinity stress on the growth and productivity of avocado by improving nutrient uptake. *A. nodosum* extract-treated avocado plants showed higher content of Ca^2+^ and K^+^ (Bonomelli et al., 2018). Further, ANE also improved the growth of turf grass grown under salinity stress by maintaining a higher K^+^/Na^+^ content (Elansary et al., 2017).

An ethyl acetate fraction of an *A. nodosum* extract (EAA) reportedly induced salinity tolerance in Arabidopsis. To further investigate the mode of action of ANE in mitigating stress, Jithesh et al. (2018) carried out a study of the global transcriptomics of EAA-treated Arabidopsis grown under salinity stress. This study showed that EAA induced the expression of 184 genes on Day 1 of treatment, further increasing to 257 genes expressed on Day 5, while 91 and 262 genes were down-regulated on Days 1 and 5 post-treatment, respectively. Similarly, Goñi et al. (2016) also compared the transcriptome of Arabidopsis treated with two different extracts of *A. nodosum*: both prepared at a temperature greater than 100°C, differing in pH and preparation method (one extract had a neutral pH while the other extract was alkaline). The application of these two different extracts induced the expression of a plethora of genes involved in stress tolerance mechanisms (Goñi et al., 2016). Both studies showed the induction of different late embryogenesis abundant (LEA) proteins and dehydrins in Arabidopsis treated with ANE. Thus, it is evident that various bioactive components of an *A. nodosum* extract were able to mitigate salinity stress through various mechanisms: by protecting cellular structures from water loss, *via* acting as a hydration buffer, sequestering ions, directly protecting other proteins or by re-naturing unfolded proteins through increased expression of LEAs (Wise and Tunnacliffe, 2004; Goyal et al., 2005; Jithesh et al., 2018).

The molecular and cellular responses of plants to salinity stress include perception, signal transduction to the cytoplasm and nucleus, gene expression, and, finally, metabolic alterations leading to stress tolerance (Agarwal et al., 2006). Salinity stress signals are first perceived by signaling molecules such as ABA and Ca^2+^, and these molecules start a cascade of events eventually leading to stress tolerance in plants (Chinnusamy et al., 2004; Bhatnagar-Mathur et al., 2008; Agarwal et al., 2013). Arabidopsis treated with an ethyl acetate fraction of ANE (EAA) and grown under salinity stress showed a higher transcript accumulation of *SnRK2*, a gene involved in the activation of the ABA-signaling network (Coello et al., 2011; Jithesh et al., 2018). Further, EAA treatments induced genes involved in ABA-dependent signaling pathways (Jithesh et al., 2018). Transcription factors (TFs) regulate the expression of various downstream target genes by interacting with the *cis*-acting element in promoters of respective target genes (Yamaguchi-Shinozaki and Shinozaki, 2006; Agarwal and Jha, 2010; Agarwal et al., 2013). The bioactive component present in *A. nodosum* extracts has been shown to regulate the convergence and interaction of various TFs such as DREB/CBF, COR47, NF-YA, COR15A, AGF2, CCA1, and LHY1, which confer stress tolerance on plants (Agarwal and Jha, 2010; Todaka et al., 2012; Goñi et al., 2018; Jithesh et al., 2018). ANE regulated both post-transcriptional as well as post-translational regulation of stress-responsive TFs (Shukla et al., 2018a). The application of ANE down-regulated the expression of miR396a-5p, which resulted in a reduction in the expression of its target gene *AtGRF7* (Yang et al., 2009; Shukla et al., 2018a). In Arabidopsis, *AtGRF7* down-regulated the expression of *AtDREB2a* by binding to its promoter element, which, in turn, acted as a down-regulator for salinity tolerance (Shukla et al., 2018a). Lower levels of *AtGRF7* in the ANE-treated plants under salinity stress led to a higher expression of *AtDREB2a* and *AtRD29* (Shukla et al., 2018a). Similarly, ANE reduced the expression of miR169 in plants grown under salinity stress conditions. The miR169 plays an important role in stress-induced flowering in plants, targeting TF NFYA (Xu et al., 2014). The application of ANE to plants grown under salinity stress delayed the induction of ath-miR169g-5p and showed a higher expression of *AtNFYA1*. This suggests that benefits of the application of ANE, as a salinity stress mitigation strategy, were due to the partial control of miR169 over *NFYA1* expression (Zhao et al., 2009; Li et al., 2013; Jithesh et al., 2018; Shukla et al., 2018a).

Salinity stress leads to the generation of reactive oxygen species (ROS) in plants, which is a well-known cause of damage to proteins, lipids, carbohydrates, and DNA, resulting in oxidative stress, which ultimately results in negative effects on plant development and growth (Mittler et al., 2004; Gill and Tuteja, 2010; Karuppanapandian et al., 2011). Salinity stress-induced production of ROS damages cell membranes by changing the saturation pattern through increased lipid peroxidation (Gossett et al., 1994; Jain et al., 2001; Miller et al., 2010). ANE application has been reported to reduce the effects of ROS generated by salinity stress in turf grass by reducing lipid peroxidation through higher activity of antioxidative enzymes. The various bioactive components of *A. nodosum* extracts reduced salinity-induced oxidative damage by eliciting the expression of glutathione *S* transferase in Arabidopsis (Jithesh et al., 2018). ANE application also alleviated oxidative damage by modulating the expression of ath-miR398, regulating the expression of its target gene, copper/zinc SOD (*AtCSD1*) (Shukla et al., 2018a).

The application of ANE had a significant influence on the expression of genes involved in the biosynthesis and transportation of flavonoids, which protect plants from ROS-induced oxidative damage during salinity stress (Jithesh et al., 2018). In addition to the regulation of regulatory genes, it was reported that ANE applications also regulated the expression of genes involved in the biosynthesis of carbohydrates (starch, sucrose, raffinose), amino acids (proline, isoleucine), and sugar alcohols (inositol, trehalose) (Jithesh et al., 2018). ANE-treated plants accumulated higher proline tissue levels under saline conditions (Elansary et al., 2017). Higher proline levels can mitigate salinity stress by stabilizing sub-cellular structures and scavenging free radicals while also buffering cellular redox potentials (Ashraf and Harris, 2004; Ashraf and Foolad, 2007; Shukla et al., 2015). Salinity stress reduces osmotic potential and affects water availability, causing physiological drought in plants. Sugar accumulation maintains total osmotic potential in plant cells during salt stress (Shukla et al., 2011). In addition to their role in osmotic adjustments, the availability and inter-organ transport of sugars play an important regulatory role in salt-stressed plants (Hare et al., 1998). The data reported by Elansary et al. (2017) indicated that ANE treatments enhanced the total non-structural carbohydrates in turf grass exposed to prolonged salinity, by increasing photochemical efficiency. ANE regulated the expression of genes involved in the metabolism and transport of carbohydrates; thus, unspecified bioactive compounds present in ANE must supply enough carbon and energy to the plant during stressful conditions.

**FIGURE 2:**
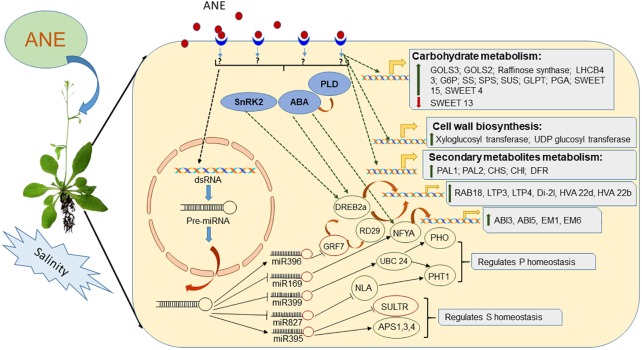
Depiction of mode of action of *Ascophyllum nodosum* extract (ANE) in mitigating salinity stress.

*Ascophyllum nodosum* treatments were also reported to improve nutrient uptake in plants grown under salinity stress. Supplementation of ANE in growth media deprived of phosphorus (P) improved its uptake and homeostasis in salt-stressed Arabidopsis by modulating the expression of miRNA399, altering the expression of its target gene *AtUBC24.* In addition to miR399, the ANE treatment also modulated the expression of miR827 and miR2111b, indicating that some components of ANE and their utilization by plant tissues have the ability to improve P-uptake in salt-stressed plants (Shukla et al., 2018a). Similarly, ANE treatments improved the architecture of Arabidopsis root systems when grown under conditions combining phosphorus deprivation and salinity stress. ANE therefore played an important role in sulfur (S) homeostasis in salt-exposed Arabidopsis by modulating the expression of miR395 (Shukla et al., 2018a). In addition to the regulation of sulfur homeostasis, ANE treatments also regulated the expression of *SULTR1;2* and *SULTR3;1* in plants grown under both normal and saline conditions (Goñi et al., 2016; Shukla et al., 2018a). Thus, ANE prevented the root tip and its meristematic cell from the injurious consequence of both stresses by regulating the expression of regulatory RNAs and genes involved in the efficient relocation of P and S resources (Shukla et al., 2018a). A clear, beneficial role for ANE has been observed in mitigating salinity stress due to its ability to improve a plant’s response to stress, both at the molecular and at the physiological level, as represented in Figure 2.

### ANE Mitigates Drought Stress in Plants

**FIGURE 3:**
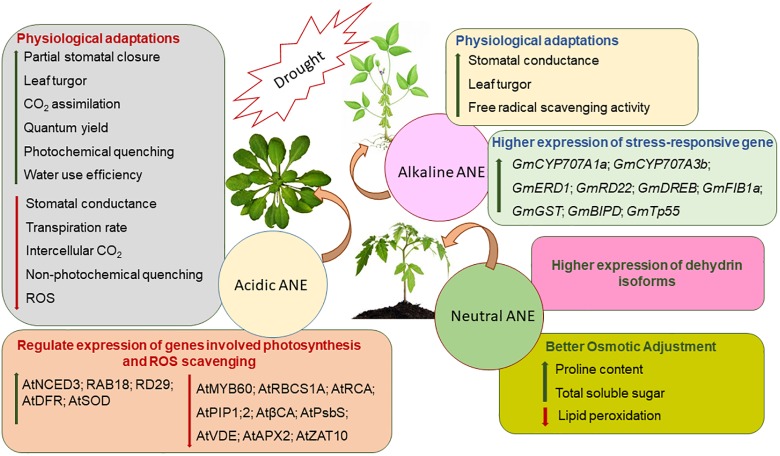
The proposed modes of action of three fractions of *Ascophyllum nodosum* extract (ANE): acidic, neutral, and alkaline ANE when applied to plants exposed to drought stress.

Both physical and physiological drought negatively impact plant physiology and thereby crop productivity by impeding nutrient and water relations, photosynthesis, and assimilate partitioning (Fahad et al., 2017; Shukla et al., 2018b). It is estimated that nearly 50% of agricultural crops are affected by drought stress worldwide (Farooq et al., 2009; Bodner et al., 2015; Joshi et al., 2016). Notable progress has been made to mitigate drought stress by using bioactive substances from *A. nodosum* (Figure 3). Several studies clearly demonstrated that the application of different ANEs alleviated drought stress in soybean (*Glycine max*), bean (*Phaseolus vulgaris*), *A. thaliana*, tomato (*Lycopersicon esculentum*), sweet orange (*Citrus sinensis*), spinach (*Spinacea oleracea*), *Spiraea nipponica*, and lemon wood (*Pittosporum eugenioides*) (Spann and Little, 2011; Xu and Leskovar, 2015; Elansary et al., 2016b; Santaniello et al., 2017; Carvalho et al., 2018; Goñi et al., 2018; Shukla et al., 2018b). The bioactive compounds (not yet fully elucidated) present in *A. nodosum* extracts when applied to stressed plants have reduced the deleterious effects of drought stress by regulating a series of sequential molecular, cellular, and physiological responses including the modulation of several genes, resulting in an accumulation of various osmolytes, an improved antioxidant system, and enhanced gaseous exchange through stomatal regulation.

Drought stress reduces transpirational cooling, therefore increasing leaf temperature (Yordanov et al., 2000). Acadian^®^, an alkaline commercial extract of *A. nodosum*, was shown to help soybean plants withstand severe drought conditions by regulating leaf temperature, turgor, and several stress-responsive genes (Martynenko et al., 2016; Shukla et al., 2018b). Stomatal conductance is a key variable of a plant’s physiological process that is influenced during drought stress (Manavalan et al., 2009). Acadian^®^ extract-treated plants showed higher stomatal conductance under drought stress (Shukla et al., 2018b), while in another study, an acidic extract of *Ascophyllum* also resulted in a reduction of stomatal conductance by down-regulating the expression of *AtPIP1*;2 and *βCA1*, key genes involved in the regulation of CO_2_ diffusion within the mesophyll (Santaniello et al., 2017). Stomatal conductance and ABA concentrations are co-related during drought stress (Manavalan et al., 2009). In drought-stressed soybean, the application of an alkaline ANE extract (Acadian^®^) modulated the expression of genes involved in the catabolism of ABA by regulating the expression of *GmCYP707A1a* and *GmCYP707A3b* (Shukla et al., 2018b). In addition, priming the plants with acid-extracted ANE induced a partial stomatal closure by down-regulating the expression of *AtMYB60*, which is known to be involved in the regulation of stomatal movement (Santaniello et al., 2017). The presence of ABA negatively regulated the expression of *AtMYB60* during drought stress. Thus, ANE-treated plants, under drought stress, induced ABA biosynthesis by boosting the expression of *AtNCED3*, which resulted in partial stomatal closure for greater water-use efficiency (Santaniello et al., 2017). In addition, ANE treatment also induced the expression of ABA-responsive genes such as *AtRAB18* and *AtRD29* in response to drought stress (Santaniello et al., 2017). Taken together, these findings suggested that alkaline-extracted ANE has different modes of action in mitigating drought stress, as compared to acid-extracted ANE. Alkaline ANE regulated stomatal conductance in an ABA-independent manner while acid-extracted ANE promotes an ABA-dependent stomatal closure during drought stress.

Drought-induced stomatal closure leads to a reduction in CO_2_ availability, directly reducing the rate of photosynthesis (Chaves et al., 2003, 2009). Treatment with the various *A. nodosum* extracts modulated photochemical efficiencies, water-use potential, and stomatal conductance of Arabidopsis, spinach, *S. nipponica*, and *P. eugenioides* (Xu and Leskovar, 2015; Elansary et al., 2016b; Santaniello et al., 2017). Acidic-extracted ANE protected Arabidopsis from drought stress by inducing partial stomatal closure, thereby preventing water loss due to transpiration. Furthermore, acid-extracted ANE protected the photosynthetic apparatus by reducing the expression of *AtRBCS1A* and *AtRCA*, which catalyze Rubisco activation during photosynthesis (Demirevska et al., 2008; Santaniello et al., 2017). The alkali process extract of *A. nodosum* regulated the expression of *GmFIB1a* and protected photosystem II (PSII) from drought-induced damage (Shukla et al., 2018b). *GmFIB1a* functioned in an ABA-dependent manner and was involved in photo-protection during stress (Yang et al., 2006). Thus, in soybean, alkali-processed ANE extract regulated both ABA-dependent and ABA-independent pathways for conferring drought tolerance (Shukla et al., 2018b).

Plants under drought conditions tend to produce ROS that include superoxide, hydroxyl, perhydroxy, and alkoxy radicals (Mittler, 2002; Farooq et al., 2009). These ROS entities are known to damage cellular constituents such as DNA, proteins, membranes, and lipids (Fu and Huang, 2001). Drought-induced ROS production results in the peroxidation of the PUFAs found in biological membranes (Fu and Huang, 2001; Jiang and Huang, 2001). The MDA (malondialdehyde) content of tissues can be used as an indicator of the extent of drought-induced peroxidative damage (Shukla et al., 2011). As an adaptive mechanism in response to drought, plants detoxify ROS by enzymatic and non-enzymatic pathways (Apel and Hirt, 2004; Baxter et al., 2014). Enzymatic ROS scavenging mechanisms in plants include SOD, APX, glutathione peroxidase (GPX), and catalase (CAT) (Miller et al., 2010). ANE applications were reported to improve drought tolerance by reducing ROS-induced MDA production in the bean (*P. vulgaris*) by improving CAT activity (Carvalho et al., 2018). Similarly, a foliar spray of ANE reduced lipid peroxidation in *Paspalum vaginatum* that was grown under prolonged irrigation (Elansary et al., 2017). Reduced ROS in ANE-treated *P. vaginatum* grown under drought stress was ascribed to increased activity of antioxidative enzymes such as SOD, CAT, and APX, and the higher production of non-enzymatic antioxidants, such as ascorbates (Elansary et al., 2017).

Proline is an important osmolyte and a signaling molecule in plants, and is credited for its role in ROS scavenging as well as in osmotic adjustment (Valliyodan and Nguyen, 2006). However, it is not clear whether proline accumulation is a symptom of stress, a response to stress, or an adaptive strategy (Carillo, 2018). Regardless, proline plays an integral role in drought adaptation by buffering cellular redox potential, stabilizing membranes and proteins, and inducing the expression of stress-responsive genes (Singh et al., 2015; Carillo, 2018). ANE was found to improve proline biosynthesis in *P. vulgaris* grown under drought stress (Carvalho et al., 2018). Similarly, a soil treatment of ANE on *S. nipponica* and *P. eugenioides* reportedly ameliorated drought stress by increasing the accumulation of antioxidants and lipid peroxidation, thus reducing the ROS content and inherent stresses (Elansary et al., 2016b). Goñi et al. (2018) showed that extracts of the same *Ascophyllum* raw material, prepared by different extraction methods, regulated drought stress in treated tomato in different ways. ANE manufactured using a proprietary process at temperatures greater than 100°C and an alkaline pH was more efficient in mitigating drought stress in *L. esculentum* (by increased antioxidants, proline, and soluble sugar accumulation) as compared to ANE manufactured at the same temperature (*T* > 100°C) but at a neutral pH (Goñi et al., 2018). Dehydrins are produced by plants in response to drought, acting as intracellular stabilizers, upon targets in both the nucleus and cytoplasm (Tommasini et al., 2008). Besides the accumulation of proline and soluble sugars in ANE-treated tomato plants, ANE treatments also induced the expression of different dehydrin-like proteins under drought stress. Together, these findings verified that various extracts from *A. nodosum* mitigated the severity of drought stress by regulating intrinsic molecular and biochemical processes in plants.

### ANE Mitigates Freezing Stress in Plants

Nearly 42% of all global land experiences temperatures below −20°C, and plants growing in these regions experience freezing stresses during periodic exposure to temperatures below 0°C (Chinnusamy et al., 2007; Miura and Furumoto, 2013). Freezing stress adversely affects plant growth and development, limiting agricultural productivity (Miura and Furumoto, 2013). During freezing stress, intracellular and extracellular ice are formed, which disrupts the integrity of cells, causing death (Burke et al., 1976; Weiser et al., 1976). Most temperate crops have an inherent tendency to acquire tolerance to low temperatures by a process known as cold acclimation, while tropical and sub-tropical plants are sensitive to low-temperature stress (Chinnusamy et al., 2003). Several studies reported that the bioactive compounds present in various types of extracts from *A. nodosum* can mitigate low-temperature stress in plants. The application of ANE on winter barley improved winter hardiness and increased frost resistance (Burchett et al., 1998). Rayirath et al. (2009) showed that the lipophilic fraction of an *A. nodosum* extract improved tolerance of *A. thaliana* grown under freezing conditions. Under control conditions, the *A. thaliana* plants grown at −5.5°C showed significant chlorosis and tissue damage, whereas plants treated with the lipophilic fraction of ANE recovered from freezing-induced damage (Rayirath et al., 2009). This study also revealed that ANE application reduced freezing-induced electrolyte leakage by maintaining membrane integrity during freezing stress. ANE also induced the expression of cold-responsive genes such as *COR15A*, *RD29A*, and *CBF3* (Rayirath et al., 2009). In order to further understand the mode of action of ANE in mediating freezing tolerance in plants, Nair et al. (2012) carried out global transcriptome and metabolome analysis of the lipophilic fraction (LPC) of ANE-treated plants exposed to −2°C. Global transcriptome analysis revealed that the LPC of ANE altered the expression of 1,113 genes in response to freezing stress. Most of these genes were found to be involved in responses to stress, sugar accumulation, and lipid metabolism. In response to freezing stress, plants tend to accumulate proline by simultaneous up-regulation of genes involved in proline biosynthesis (*P5CS1*, *P5CS2*) and down-regulation of genes involved in proline catabolism (*ProDH*). Application of the LPC fraction of ANE increased the proline content in response to freezing stress by modulating the expression of *P5CS1*, *P5CS2*, and *ProdH* (Nair et al., 2012). Therefore, ANE improved freezing tolerance in plants by inducing proline biosynthesis.

Metabolite profiling of the LPC fraction of ANE-treated Arabidopsis plants revealed that protection was achieved by regulating pools of soluble sugars, sugar alcohols, organic acids, and lipophilic components such as fatty acids (Nair et al., 2012). Sugar accumulation helps plants overcome freezing stress by playing an important role in stabilizing various biological components such as the cellular membrane and membrane-bound organelles (Tarkowski and Van den Ende, 2015). The LPC of ANE failed to improve freezing tolerance in the *SFR4* mutant of Arabidopsis, which is known to be defective in the accumulation of free sugars (Nair et al., 2012). These results suggested that an ANE treatment, prior to freezing stress exposure, induced the accumulation of soluble sugars. These results provided evidence to support the claim that ANE plays an important role in improving freezing tolerance in plants through molecular, biochemical, and physiological changes.

## ANE Improves Plant Defenses Against Various Pathogens

Changing climatic conditions and intensive agricultural practices increase the emergence of infectious plant diseases, causing a reduction in agricultural productivity (Anderson et al., 2004; Ayliffe and Lagudah, 2004). Plant diseases are caused by pathogens such as bacteria, fungi, and viruses (Pieterse and Dicke, 2007; Stadnik and Freitas, 2014) that disrupt plant health as well as their productivity. Plants have evolved several inducible defense mechanisms in order to deter these pathogens following infection (Conrath et al., 2002; Wiesel et al., 2014). Two types of disease resistance mechanisms in plants have been reported: systemic acquired resistance (SAR) and induced systemic resistance (ISR). In SAR, SA plays a crucial role of mediating pathogenesis-related (PR) gene activation, while in ISR, JA, and ethylene (ET) pathways are important for the induction of broad-spectrum disease resistance (Gaffney et al., 1993; van Loon et al., 1998). Elicitors are defined as compounds of biological origin capable of inducing defense responses in plants (Conrath et al., 2002; Wiesel et al., 2014). Elicitors are molecules such as lipo-polysaccharides, chitin, and bacterial flagella. Furthermore, some synthetic chemicals, e.g., chitosan, 2,6-dichloro-isonicotinic acid, β-aminobutyric acid, methyl jasmonate, and benzothiadiazole, have also been reported for their ability to induce SAR and ISR against various plant pathogens (Dixon, 2001; Mercier et al., 2001; Bektas and Eulgem, 2015; Iriti and Varoni, 2015).

Over the course of evolution, various seaweeds have developed efficient defense mechanisms in order to fight their own natural pathogens (Potin et al., 1999; Shukla et al., 2016). Less incidence of pathogen infection is seemingly observed in seaweeds because they are rich sources of unique bioactive compounds such as fucans, carrageenans (e.g., *i*, *k*, and λ), ulvans, and laminarins (or fucose containing polymers) (Klarzynski et al., 2003; Sangha et al., 2010; Vera et al., 2011). These seaweed-based bioactive compounds are known to induce defense responses against pathogens by acting as priming or elicitor molecules (Khan et al., 2009; Sharma et al., 2014; Shukla et al., 2016). These elicitors act as pathogen-associated molecular patterns (PAMPs) (Sharma et al., 2014). PAMPs bind to host *trans*-membrane pattern recognition receptors (PRRs) and prime the plants by inducing ISR and SAR responses (Eckardt, 2008; Zipfel, 2009). Primed plants induced a greater preventative response against the progression of the pathogen infection as compared to unprimed plants.

It was reported that bioactive compounds present in ANE elicited defense responses against various pathogens (Patier et al., 1995; Sharma et al., 2014). Marmarine (IFTC^TM^, Amman, Jordan), a commercial extract of *A. nodosum*, improved plant defense against *Phytopthora melonis* in cucumber (Abkhoo and Sabbagh, 2016). The application of the extract [30 ml per plant, 0.5 or 1% Marmarine, alternating with 2 g L^−1^ of fungicide (metalaxyl), applied to 21-day-old seedlings through root drench and/or foliar spray at 5-day intervals for a total of three applications] led to enhanced activation of disease resistance enzymes including peroxidase, polyphenol oxidase, lipoxygenase, phenylalanine ammonia lyase, and β-1,3-glucanase. This work highlighted the role of certain seaweed extracts on different plant enzymes and genes that could result in the induction of defense mechanisms (or disease resistance) in cucumber (Abkhoo and Sabbagh, 2016). Similarly, Panjehkeh and Abkhoo (2016) revealed that the same initial application of *A. nodosum* extract Dalgin [Sustainable Agro Solutions (SAS), Spain] alternating with 2 g L^−1^ of fungicide (metalaxyl), as in the Abkhoo and Sabbagh (2016) study (30 ml per plant, 0.5 or 1% Dalgin, applied to 21-day-old seedlings), was able to induce resistance (ISR) against *Phytophthora capsica*, a fungal disease in tomato. Similarly, the alternating application of Stimplex^®^, a liquid-based extract of *A. nodosum* with fungicide (chlorothalonil, 2 g L^−1^), reduced the progression of fungal disease in cucumber through the induction of defense genes and enzymes (Jayaraman et al., 2011).

The mechanism of *A. nodosum* extract-induced resistance in *A. thaliana* against *Pseudomonas syringae* pv. tomato DC3000 was carried out by Subramanian et al. (2011). Different extracts from *A. nodosum* induced resistance in SA-deficient plants, while extracts did not elicit an effect on *JAR1* (jasmonic acid resistance 1) mutant. In addition to this, the application of ANE induced the expression of JA-related genes such as *PDF1.2*, while expressions of *PR1* and *ICS1* were not greatly affected by ANE (Subramanian et al., 2011). These results suggested that ANE induced resistance in challenged Arabidopsis by activating the JA-dependent signaling pathway. Different solvent fractions exhibited reduced development of disease symptoms on the leaves, which is correlated with the increased expression of jasmonic-acid-related gene transcripts (Subramanian et al., 2011). *Kappaphycus* and *Eucheuma* spp., economically important red algae, were reported to be susceptible to epiphyte infestations (Loureiro et al., 2012). It was reported that a dip application of ANE (as a soluble seaweed extract powder, given the acronym AMPEP—*Ascophyllum* Marine Plant Extract Powder) elicited a natural defense mechanism in cultivated *Kappaphycus* against the epiphytes *Neosiphonia apiculata*, *Cladophora*, and *Ulva*, by inducing the phenolic content, free-radical scavenging, and iron chelation (Loureiro et al., 2010, 2012; Hurtado et al., 2012; Ali et al., 2018).

**FIGURE 4:**
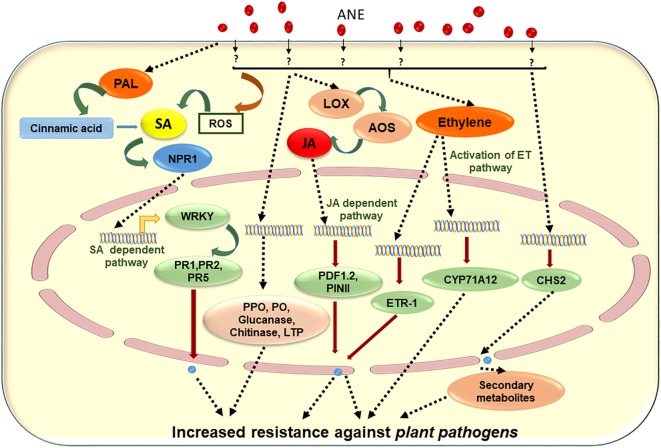
Schematic representation of proposed mode of action of *Ascophyllum nodosum* extract (ANE) in eliciting plant defense against different plant pathogens.

Another *A. nodosum*-derived extract (Stella Maris^®^) was reported to boost plant immunity by elevating the production of hydrogen peroxide, which ultimately led to an increase in the concentration of ROS. It was further shown that the expression of plant immune response genes *WRKY30*, *CYP71A12*, and *PR-1* (genes that activate in early, mid, and late phases of immunity in the plant, respectively) was up-regulated (Cook et al., 2018). The priming of 3-week-old *A. thaliana* plants with 1 g/L of ANE (25 ml per plant through root drench) 2 days prior to inoculation protected against the necrotic pathogen, *Sclerotinia sclerotiorum* (Subramanian et al., 2011). Similarly, Jayaraj et al. (2008) showed that a foliar spray of ANE to carrot plants significantly reduced the progression of disease caused by *Alternaria radicina* and *Botrytis cinerea*. It was found that the priming of carrot plants with ANE induced the activity of defense-related enzymes including peroxidase (PO), polyphenoloxidase (PPO), phenylalanine ammonia lyase (PAL), chitinase, and β-1,3-glucanase, as well as increasing the transcript accumulation of *PR-1*, *PR-5*, *NPR-1*, *LTP*, chalcone synthase, and PAL. Based on the available literature, Figure 4 was prepared to depict elicitors present in ANE, which are known to improve plant defense responses against different pathogens. The published evidence suggested that judicious applications of extracts from ANE could be an effective tool in disease management (Table 3). This strategy minimizes the use of chemical-based fungicides and provides an environmentally safe and sustainable method for the management of plant diseases.

**Table 3:** List of the different extracts from *A. nodosum* inducing disease resistance in different plants against different pathogens.

S. No.	Extract	Crop	Causal organism	Disease	Function	References
1	Maxicrop^®^ Triple	Strawberry	*Tetranychus urticae*	–	Reduces the population of two-spotted red spider mites on treated plants	Hankins and Hockey, 1990
2	Maxicrop^®^ Original	Arabidopsis	*Meloidogyne javanica*	Root-knot	Reduces number of females of *M. javanica*	Wu et al., 1998
3	*A. nodosum* extract (Acadian Seaplants)	Carrot	*Alternaria radicina* and *Botrytis cinerea*	Black rot, Botrytis blight	Induces expression of defense related genes or proteins	Jayaraj et al., 2008
4	Stimplex^®^ (Acadian Seaplants)	Cucumber	*Alternaria cucumerinum*, *Didymella applanata*, *Fusarium oxysporum*, *Botrytis cinerea*	Alternaria blight, Gummy stem blight, Fusarium root and stem rot, Botrytis blight	Stimplex reduces the disease by activating different-related enzymes and accumulation of secondary metabolites	Jayaraman et al., 2011
5	*A. nodosum* extract (Acadian Seaplants)	*K. alvarezii*	*Polysiphonia subtilissima*	Ice–ice, goose bumps	Reduces the growth of the epiphyte	Loureiro et al., 2010
6	*A. nodosum* extract (Acadian Seaplants)	Arabidopsis	*Pseudomonas syringae*, *Sclerotinia sclerotiorum*	Bacterial speck, stem rot	Reduces the development of diseases, which is correlated with expression of jasmonic-acid-related gene transcript	Subramanian et al., 2011
7	Marmarine	Cucumber	*Phytophthora melonis*	Damping-off	Induces defense-related enzymes	Abkhoo and Sabbagh, 2016
8	*A. nodosum* extract (Acadian Seaplants)	Tomato	*A. solani*, *X. campestris* pv *vesicatoria*	Alternaria blight; bacterial leaf spot	Reduces incidence of diseases in plants by the upregulation of JA/ethylene pathway	Ali et al., 2016
9	AMPEP	*K. alvarezii*	*Neosiphonia apiculata*	Ice–ice	Reduces the biotic stress caused by endophytes	Ali et al., 2018
10	Dalgin^®^	Tomato	*Phytophthora capsici*	Damping-off	Induces expression of defense-related genes or proteins	Panjehkeh and Abkhoo, 2016
11	Stella Maris^TM^	*Arabidopsis thaliana*	*Pseudomonas syringae DC3000*, *Xanthomonas campestris BP109*		Inhibited the growth of multiple bacterial pathogens by inducing the expression of WRKY30, CYP71A12 and PR-1 gene	Cook et al., 2018

## ANE Improves Soil Health

Soil health, alternatively known as soil quality, is simply defined as: “the continued capacity of soil to function as a vital living ecosystem that sustains plants, animals and humans” (U.S. EPA, 2012). A healthy soil contributes to environmental management within the biosphere (air, water, and soil) and the productivity of plants and animals under both natural and managed systems (Karlen et al., 1997; Doran and Zeiss, 2000). Soils need improvement in order to enhance their ability to sustain their environmental and biological purposes. Select seaweed extracts have been studied sufficiently to suggest that their use as agricultural inputs have two modes of action: (1) they are biostimulants, as discussed above, that enhance growth and productivity of crop plants, and (2) they are chelators, directly contributing to the health of the soil (Khan et al., 2009). ANE provides natural chelation in the soil due to the presence of residual alginates present in the hydrolyzed extract, which allows for an increase in plant-available minerals and increased soil aeration and water-holding capacity (Spinelli et al., 2010; Craigie, 2011; du Jardin, 2015; Illera-Vives et al., 2015). Actiwave^®^, a metabolic enhancer prepared from *A. nodosum*, was used as a natural iron chelator for improved productivity of strawberry (Spinelli et al., 2010).

Alginic acid is a polysaccharide made up of mannuronic and guluronic acid units derived from brown seaweeds (Yabur et al., 2007; Craigie, 2011; Battacharyya et al., 2015). Alginic acids are a major constituent of the algal cell wall, comprising between 15 and 30% by dry weight (Yabur et al., 2007; Khan et al., 2009; Craigie, 2011; Battacharyya et al., 2015). Once commercially extracted, alginates are able to form natural gums or gels based on their composition (i.e., ratio of M:G, mannuronic acid:guluronic acid) and through their ability to bind water (Glicksman, 1987; Yabur et al., 2007). Alginates have been found to improve the physical conditions of soil (Khan et al., 2009; Illera-Vives et al., 2015). Through natural chelation, alginates bind to metal ions in the soil forming complex polymers (i.e., high molecular weight), and these molecules absorb moisture and swell as a result (Khan et al., 2009; Battacharyya et al., 2015). It is these swollen molecules that increase soil aeration and water-holding capacity (Khan et al., 2009; Spinelli et al., 2010). Further, through the aforementioned process, the presence of alginate in the rhizosphere alters the soil structure to become a more conducive environment for plant and microbial growth activity (Battacharyya et al., 2015).

### Change in the Host Plant Induces Change in the Rhizospheric Microbial Population

The interaction between soil microbes and plants is cyclic in nature, known loosely as soil community feedback (Bever et al., 2012). The composition of the soil microbial population is based on the presence of the plant roots in the soil and compounds in the soil. Plants will grow with the help of molecules in the soil provided, in part, by the soil microbial population (Bever et al., 2012). There are interactions between plant roots (inter- and intra-species), between plant roots and insects, and between roots and rhizospheric microbes (Bais and Kaushik, 2010). Furthermore, there are also complex interactions between the aforementioned microbes, insects, and roots with root exudates (Bais and Kaushik, 2010).

The pretreatment of 10-day-old *Medicago sativa* (alfalfa) plants with 1 g/L of Acadian^®^ (Acadian Seaplants Limited, 100 ml total) 2 days prior to inoculation with *Sinorhizobium meliloti* more than doubled the number of bacteria present in the rhizosphere, 12 h post-inoculation as compared to the untreated control (Khan et al., 2012). The seaweed extract induced the plant to produce root exudates (i.e., flavonoids) that would attract the bacteria to the root surface (Khan et al., 2012). Similarly, it is also reported that the application of ANE and its organic fractions induced rhizobium nodulation by regulating the legume-rhizobia signaling process (Khan et al., 2013).

### Changes in Modes of Action and/or Function of Rhizospheric Microbial Population

The composition of the rhizospheric microbial population is dependent on a plethora of factors, including soil temperature, water-holding capacity, oxygen supply, and soil cultivation practices (i.e., history of fertilizer and pesticide applications and tillage) (Kilian et al., 2000). A change in any one of these factors could significantly impact the composition of the various microbial populations as well as the microbial functionality in the soil (Kilian et al., 2000). The application of select seaweed extracts directly to the soil or indirectly to the plant has also been reported to alter the rhizospheric microbial population.

A soil drench application of Actiwave^®^ (10 ml of extract in 20 ml of water per plant) on strawberry plants increased the rhizospheric microbial population (Spinelli et al., 2010) and subsequent metabolic activity when applied in lower concentrations, compared to untreated soils as a result of stimulation from the bioactive components in the extract (Alam et al., 2013). The root-drench application of an *Ascophyllum* extract improved the growth of strawberries and carrots by acting as a prebiotic and increasing soil microbial activity (Alam et al., 2013, 2014).

Conversely, constituents in various seaweed extracts have shown effectiveness as biocontrol agents against bacteria, viruses, fungi, and nematodes (Nabti et al., 2017). A soil drench application of an alkaline seaweed extract (Maxicrop Original^®^, Maxicrop International Limited) to the soil of *A. thaliana* plants significantly reduced the number of deleterious female nematodes (*Meloidogyne javanica*) and number of eggs, compared to untreated soils (Wu et al., 1998). This study and others (Wu et al., 1998) suggested that the betaine constituent of the extract was responsible for inducing a defense reaction in *A. thaliana* and *L. esculentum* (tomato) against the root-knot nematode (Wu et al., 1998).

## Conclusion and Future Challenges

In the current agricultural landscape, cultivation practices are reliant on synthetic chemicals [approximately 200 teragrams (1 Tg = 10^12^ g) per year worldwide] (Wu et al., 2018) to combat abiotic and biotic stresses (pesticides) and to promote plant growth (fertilizers). The short- and long-term negative impacts of synthetic chemicals on the environment and associated plant and animal health are becoming more prevalent every day. However, to sustain the growing human population, agriculture must be more productive than ever, with less viable resources and variable growing conditions (i.e., cultivatable soils, access to water and nutrients, consistent temperatures, etc.). To reduce reliance on synthetic chemicals, the solution must include multiple sources of natural compounds that are proven to promote crop growth under seemingly inadequate growing conditions and inherently refuel the surrounding ecosystems with more beneficial compounds, i.e., perform the roles of the pesticides and fertilizers without the harmful side effects. The utility of various extracts of *A. nodosum*-based products as biostimulants is multi-faceted: this complex alga and its extracts have shown efficacy in promoting plant growth and improving crop plant resilience to environmental perturbations, while being a natural, marine species, and therefore, when applied correctly (i.e., defined rates and timings of applications), they pose no harmful effects. Furthermore, ANE has been reported to act as both a biocontrol agent and a soil-microbial supplement.

Although the existing evidence for *A. nodosum* extracts as biostimulants in agriculture is promising, moving forward, it is important to focus the research in order to fully saturate agricultural practices with these extracts. Now that we are beginning to accumulate evidence on the modes of action of the extracts, we need to evaluate other aspects of extract application to optimize the desired mode of action. This push for more information creates a plethora of research questions: What is the optimal application rate of ANE, and in what application method (i.e., drench or spray)? When is the optimal time of application, and is there need for re-application during the growing season? If so, at what time intervals? How do these answers vary between crops and between climatic locations? Additionally, there are differences between extracts of *A. nodosum* based on extraction method and the resultant composition of the extract. How can current extraction methods be optimized to reap the most benefits from each extract? Can extraction methods not previously used with *A. nodosum* be adopted industrially (i.e., E-AE)? How do the resultant extracts compare to currently available (and reasonably well studied) extracts, and how can we exploit their positive modes of action? Furthermore, it is important to investigate whether different modes of application inherently alter the mode of action of the extracts in improving plant growth through the integration of modern interdisciplinary science. The application of the research to real-world producers will be of great benefit to understand any changes in behavior of the extracts under environmental conditions, while further identifying the modes of action will increase the extension of applications of the extracts into other fields.

## Author Contributions

PS, EM, AC, and BP conceived the layout. PS, EM, MA, SB, AC, and BP wrote the review. All authors reviewed and agreed with the final version of the submitted manuscript.

## Conflict of Interest Statement

The authors declare that the research was conducted in the absence of any commercial or financial relationships that could be construed as a potential conflict of interest.

## Footnotes

1 www.biostimulants.eu
